# Women’s response regarding timing of genital surgery in congenital adrenal hyperplasia

**DOI:** 10.1007/s12020-024-04080-z

**Published:** 2024-10-18

**Authors:** Henrik Falhammar, Gundela Holmdahl, Helena Filipsson Nyström, Anna Nordenström, Kerstin Hagenfeldt, Agneta Nordenskjöld

**Affiliations:** 1 Department of Endocrinology, Karolinska University Hospital, 171 77 Stockholm, Sweden; 2 Department of Molecular Medicine and Surgery, Karolinska Institutet, 171 76 Stockholm, Sweden; 3 Department of Women’s and Children’s Health, Center of Molecular Medicine, Karolinska Institutet, 17176 Stockholm, Sweden; 4 Department of Pediatric Surgery, Astrid Lindgren Children’s Hospital, Karolinska University Hospital, 17176 Stockholm, Sweden; 5 Institute of Medicine, Sahlgrenska Academy, University of Gothenburg, Göteborg, Sweden; 6 Department of Endocrinology, Sahlgrenska University Hospital, Göteborg, Sweden; 7 Wallenberg Center for Molecular and Translational Medicine, Göteborg, Sweden; 8 Pediatric Endocrinology Unit, Karolinska University Hospital, Stockholm, Sweden

**Keywords:** 21-hydroxylase deficiency, CAH, 46,XX, Feminizing genitoplasty, Early, Late

## Abstract

**Purpose:**

To study what adult women with congenital adrenal hyperplasia (CAH) thought about the timing of genital surgery.

**Methods:**

As part of a larger follow-up study performed between the years 2002–2005 there were questionnaires concerning genital surgery, type of surgery, their thoughts about timing of genital surgery and experience of information about surgery. Early surgery was defined as ≤4 years of age and late ≥10 years. The medical and surgical files were reviewed.

**Results:**

62 women with CAH due to 21-hydroxylase deficiency, mean age 28 years (18–63) were included. The age at first genital surgery was 3 years (0–28 years) in the 52 patients (84%) who had had genital surgery, with 60% had early surgery (≤4 years) and 29% late (≥10 years). Almost half of the cohort had a positive experience of the information about surgery, a third had no opinion and a fifth had a negative experience. Of the women 39% thought that early surgery was good, while 19% thought it should be done during or after puberty and 42% had no opinion. Of those preferring early surgery 70% had early surgery themselves. Vaginal surgery was less common among those favoring early surgery. Age, phenotype, genotype, decade of surgery and experience of the information about surgery did not differ significantly between the three groups.

**Conclusion:**

Equal numbers of women had no opinion regarding age at surgery or preferred early surgery while 19% thought it would be preferred to have surgery during or after puberty.

## Introduction

Congenital adrenal hyperplasia (CAH) is a group of autosomal recessive disorders affecting the steroid synthesis. The far most common cause of CAH is 21-hydroxylase deficiency (21OHD) [1]. In 21OHD cortisol and aldosterone concentrations are often low. In contrast, androgen and steroid precursor concentrations are high resulting in 46,XX neonates being born with variable degrees of virilized genitals. In the classic phenotypes, i.e., salt-wasting (SW) and simple virilizing (SV) CAH, the 46,XX neonates almost all have atypical genitals with variations. However, in the mild non-classic (NC) CAH most 46,XX neonates have normal genitals or just a mild clitoral enlargement [2, 3].

Children with 46,XX karyotype CAH and atypical genitals have been surgically adapted in female direction. Depending on the degree of virilization the surgical procedures have differed and included, clitoral reduction surgery (previously also clitoris amputation) and vaginal surgery. Vaginal surgery often include opening and mobilization of the common urethral and vaginal duct (i.e., the virilized urethra with insertion of the vagina on different levels) in addition to vulvoplasty with reconstruction of the introitus and labia [4]. The aims are to create normal looking feminine external genitals and possibly stimulate adequate bladder emptying with no urinary incontinence or infections. Moreover, the aims are also to permit vaginal penetration in adulthood and a normal reproductive life [4]. Traditionally, many surgeons have preferred, after parental informed consent, the genital reconstructive surgery to be performed early (2^nd^ to 6^th^ month or at least before 2 years of life). This is due to good tissue elasticity, to prevent potential hydrometrocolpos and to reduce parents’ and doctors’ distress [4, 5]. However, the timing of the genital surgery is controversial due to reported disappointing results and potential complications of surgery. Moreover, patient advocacy groups have argued that cosmetic surgery should only be performed with the individuals own informed consent [1, 6, 7]. Major institutions such as United Nations special rapporteur on torture in 2013 and European council and parliament in resolution 2191 (2017) advocate from a human rights perspective for postponing genital surgeries if not vital for health until the individual can give own informed consent. A moratorium on early genital surgery in individuals with intersex conditions has been implemented in some countries and health care institutions [3, 8].

This is a sensitive topic, difficult to assess in an informative way since patients tend to be satisfied with the treatment they have received due to positive coping strategies.

A reanalysis and investigation of previous collected data with the timing of surgery as an overarching question were performed. The aim was to assess the women’s response to the question on age for surgery in relation to their own treatment and surgical outcome regarding clitoris.

## Methods

All participating women with CAH were born 1940–1984 and examined during the years 2002–2005, as part of a larger follow-up study, including endocrine, as well as surgical aspects. Details concerning these can be found elsewhere [2, 9–14]. Among other things the women filled out a semi-structured questionnaire including questions if they had had genital surgery, type of surgery, their own thoughts about timing of genital surgery and experience of information about surgery. In English translation these questions were “How do you think the healthcare system should deal with children and women with the same illness as you have, concerning the timing of surgery? What is your experience of the information about surgery? Do you have any additional comments that you would like to pass on?”

The medical and surgical files were reviewed. All had been diagnosed with 21OHD, clinically, biochemically and genetically. All were on glucocorticoid medications and the majority also on fludrocortisone (*n* = 51, 82%). The cosmetic results including clitoris size at assessment were investigated.

Early surgery was defined as genital surgery performed at 4 years of age or earlier and late at 10 years or later to capture the majority of women with CAH that had had surgery in the current study and 10 years is approximately the median age for start of puberty in girls. The possible responses to the question on thoughts on age at surgery were divided into early, during or after puberty and no opinion. E.g., a response that the surgery should be done as early as possible was grouped into the early group, a response during teenage years/puberty was grouped into the late group, those recommending specific ages, e.g,12–13 years, was group according to that, i.e., in this case the late group. Those with a response of “I don’t know” was group in the no opinion group. The responses regarding experience of the information about surgery were divided into mainly positive, mainly negative or no opinion.

### Statistical analysis

Non-parametric statistical analysis was used. Continuous data were analyzed using Kruskal-Wallis test while categorical data were analyzed using the Chi2 test. A *p*-value < 0.05 was considered statistically significant.

## Results

In total 62 women with CAH with mean age 28 years (18–63) agreed to participate. The SW and SV phenotype were equally common (45% each) and only 10% had the NC form (Table 1). The most common genotype groups were null (23%), I2G (24%) and I172N (40%). The mean age at first genital surgery was 3 years (0–28 years) in the 52 patients (84%) who had had genital surgery, with 60% had early surgery and 29% late. Before 1 year of age 4 (8%) had had genital surgery performed and 15 (29%) at 2 years or younger. The decade of surgery was from the 1950s to the 1990s and equal numbers had had clitoral and vaginal surgery (Table 1). Almost half reported positive experience of the information about surgery while a third had no opinion and a fifth had negative experience. In the whole cohort 42% had no opinion regarding timing of genital surgery, early surgery was thought of as preferred by 39%, while 19% had the opinion that it should be done during or after puberty. Of those in favor of early surgery 70% had had early surgery themselves while among those in favor of late surgery 42% had had late surgery themselves.

**Table 1:** Basic characteristics, genital surgery, experience of the information about surgery and the woman’s own recommendation of timing of surgery in adult women with congenital adrenal hyperplasia due to 21-hydroxylase deficiency as well as dividing and comparing those recommending early vs. during or after puberty vs. no opinion timing of surgery

	All *n* = 62	Recommendation of timing of surgery			*P*-value^a^
		Early *n* = 24 (39%)	During or after puberty *n* = 12 (19%)	No opinion *n* = 26 (42%)	
Age (years)	28 (18–63)	32.5 (18–63)	30 (18–57)	28 (19–63)	0.31
Phenotype SW/SV/NC (*n*)	45%/45%/10% (28/28/6)	54%/42%/4% (13/10/1)	33%/67%/0% (4/8/0)	42%/38%/19% (11/10/5)	0.15
Genotype null/I2G/I172N/P30L/V281L/Others (*n*)	23%/24%/40%/2%/6%/5% (14/15/25/1/4/3)	25%/29%/33%/4%/4%/4% (6/7/8/1/1/1)	17%/8%/75%/0%/0%/0% (2/1/9/0/0/0)	23%/27%/31%/0%/12%/8% (6/7/8/0/3/2)	0.36
Genital surgery	84% (52/62)	96% (23/24)	100% (12/12)	65% (17/26)	0.003
Age at first genital surgery	3 (0–24)	3 (0–24)	5.5 (0–21)	4 (0–16)	<0.001
Early surgery (≤4 years)^b^	60% (31/52)	70% (16/23)	50% (6/12)	53% (9/17)	0.29
Late surgery (≥10 years)^b^	29% (15/52)	13% (3/23)	42% (5/12)	41% (7/17)	0.08
Decade of surgery 50 s/60 s/70 s/80 s/90 s (*n*)	8%/15%/23%/42%/12% (4/8/12/22/6)	4%/29%/29%/29%/4% (1/7/7/7/1)	8%/8%/8%/50%/17% (1/1/1/6/2)	8%/0%/12%/35%/15% (2/0/3/9/4)	0.40
Clitoral surgery	71% (37/52)	87% (20/23)	67% (8/12)	59% (10/17)	0.12
Vaginal surgery	79% (41/52)	61% (14/23)	92% (11/12)	94% (16/17)	0.018
Clitoris size (cm)	3 (0–5)	2 (0–5)	3 (2–5)	3 (1–3.5)	0.77
Patients’ own opinion about clitoris size					
Too large	39% (23/59)	30% (7/23)	67% (8/12)	33% (8/24)	0.087
Just right	49% (29/59)	48% (11/23)	33% (4/12)	58% (14/24)	0.36
Too small	12% (7/59)	22% (5/23)	0% (0/12)	8% (2/24)	0.13
Experience of the information about surgery					
Positive	46% (24/52)	43% (10/23)	50% (6/12)	47% (8/17)	0.93
Negative	19% (10/52)	17% (4/23)	25% (3/12)	18% (3/17)	0.90
No opinion	35% (18/52)	39% (9/23)	25% (3/12)	35% (6/17)	0.70

^a^*P*-value for comparing recommending early surgery vs. during or after puberty vs. no opinion

^b^4 patients with genital surgery had it between 5 and 9 years of age and were not included

Almost all with genital surgery had an opinion about timing, while those who had not had genital surgery only 65% had an opinion (Table 1). Those with an opinion favoring early surgery had had surgery earlier than the other two groups. Vaginal surgery was less common among those favoring early surgery. Age, phenotype, genotype, decade of surgery and experience of the information about surgery were similar between the three groups. The size of clitoris or their opinion about the size did not differ significantly between the groups. Of note, there were 8 patients who had had clitorectomy.

## Discussion

In this retrospective study of adult women with CAH, the majority had had genital surgery, 60% early and 29% late. A large proportion of the whole cohort (42%) had no opinion about the timing of surgery. Those preferring early surgery (39%) had themselves had earlier surgery more often (70%) than those preferring surgery during or after puberty (42%) or those with no opinion. Of the 10 women who did not have any surgery 9 had no opinion. It should be noted that the data was collected 20 years ago before the present discussion and questioning of early genital surgery. Hence, the women in this study have not been exposed to the discussion from a human rights perspective and the thoughts about individual self-determination.

Genital reconstructive surgery in 46,XX children with CAH and atypical genitals is complex and has become controversial [4, 15]. There is, however, mostly consensus that the decision regarding genital surgery and the timing should be done together with the parents and when possible the patient. Moreover, genital surgery should be performed after discussions in a multidisciplinary team [1, 3, 4]. Genital surgery should further only be performed in centers with experienced pediatric surgeons/urologists, pediatric endocrinologists, pediatric anesthesiologists, behavioral/mental health professionals, and social work services [15]. There is also at least to some extent consensus that surgery should be performed either early (the first year/years of life) or at puberty and also to be more restrictive in cases of mild virilization [15]. It is today more common to wait for the effect of glucocorticoid treatment when the clitoris can be assumed to be less enlarged before a decision on surgery. This will hopefully in the future add knowledge about non-surgical treatment outcome. Previously many surgeons recommended early single-stage surgery to take advantage of the early estrogen effect and use tissue from the enlarged clitoris for the vulvoplasty with the argument that this relives the anxiety in the families [5, 15]. However, a recent report showed that there is persisting concerns regarding the 46,XX child’s genitalia regardless of surgery or not [16]. The controversies have arisen since the genital surgery can be regarded as an esthetic procedure in a small child, with no benefit at that age for the child and with a risk of disturbing sensitivity in the clitoris later in life [17]. Follow-up studies have shown a high percentage of secondary surgery later in life due to functional and esthetic reasons as judged by gynecologists or the patients themselves [18, 19]. In addition, the surgery is irreversible making the decision more controversial. Patient advocacy groups have argued that all genital surgery should be delayed until the child with atypical genitalia is able to give full informed consent and participate in the decision-making [6, 7]. This has resulted in a legal ban in conducting genital surgery for 46,XX children with atypical genitals in some countries such as Germany. Genital surgery have also been condemnated by organizations such as United Nations rapporteur on torture, Amnesty International, European Union Agency for Fundamental Rights, Human Rights Watch and Council of Europe Commissioner for Human Rights [3, 8]. However, the largest group representing patients with CAH in USA, the Congenital Adrenal Hyperplasia Research Education and Support Foundation (CARES), opposes a ban on early surgery [8]. It should be noted that, in recent studies, most parents favor early surgery with very few exceptions, even to a larger extent than the women themselves [6, 8, 20, 21].

Data on what the women with CAH think regarding timing of surgery, are more scarce. In a Finnish study of 24 women (CAH *n* = 16) who had had genital surgery in childhood (median age 2.1 years, range 0.4–14.8), none thought the procedure had been performed too early and 17 believed that surgery had been done at a proper age [22]. Of note, 3 women with CAH thought the surgery had been done too late (had been performed at 9, 14 and 17 years, respectively). In a French study 21 adult women with CAH and controls were included. Of the women with CAH 90% (100% of those with early surgery and 80% of those with late surgery) considered early surgery before 1 year of age as the preferred option while only 52% of controls thought so [23]. In a Malaysian study 59 girls/women with CAH aged 10–28 years were recruited. Of these 51% did not respond to the question about timing of surgery while 36% recommended early surgery and 13% late [21]. In a UK survey study including 12 women with CAH of whom 8 had had surgery the preference was for early surgery [24]. In a pan-European study including 226 adult women with CAH only 144 responded, i.e., 36.3% probably had no opinion on the question about timing of genital surgery. Among those responding 76% favored early, 10% late and 14% were indifferent [3]. Similarly, we found in the current study that the majority of adult women with CAH with an opinion recommended early surgery. Of those with a personal opinion favoring early surgery 70% had had early surgery themselves while in those favoring late surgery 42% had had late surgery. Thus, those who had had early surgery were more prone to advocate early surgery which may be a reflection of good coping strategies. It is an inherent difficulty for this type of studies that the patients with good coping will come to terms with their situation. It should be noted though that 42% of our adult women with CAH had no opinion on the timing of the surgery. 46,XX children with CAH and Prader 4–5 are usually not separated from Prader 1–3 in similar studies, such as the current study, possibly due to a limited number of participants. It is likely that much unnecessary surgery has been performed in girls and women with Prader 1–3. Patients who have undergone genital surgery are unable to compare their experience to what it might have been like without the procedure. Very little data exist on how the girls perceive growing up with a mild to moderate clitoromegaly and how much genital surgery is needed if delaying until puberty. The results after genital surgery in puberty are likely better both esthetically and functionally. It should be noted that it is difficult to reconstruct in an area with scars and strictures. Postponing surgery is of course most difficult in Prader 3–5 and severe genetic variants where there is risk of psychological burden growing up if no genital surgery has been performed. However, we cannot be certain what these individuals would have preferred if they had been able to consent before early surgery.

Even more scarce are studies on experiences concerning living with clitoromegaly. In this group of women that were not operated, 54% regarded their clitoris as “too large” and among the operated women 66% regarded the clitoris to be of normal size [2]. Earlier studies interviewing women with CAH gave many examples of the stigma felt regarding a large clitoris [25]. Recently an on-line survey answered by 97 women with CAH reported that they recognized the clitoromegaly at a median age of 11–13 years and that there were no positive effects [26]. The condition affected many activities like sports and less wish for tight clothing or changing clothes in public locker rooms. They also experienced poor self-esteem, gender self-perception and body image.

Another argument against early surgery is the risk of future change of gender identity. However, the great majority of 46,XX children with CAH raised as girls will not develop gender dysphoria in adulthood [27]. Having said that, many will develop a non-heterosexual orientation which is associated with the severity of the genotype [13, 28].

### Limitations

Even though this was a fairly large study of adult women with CAH, the number of participants was still limited which prevented us from doing subgroup analysis of the different pheno- and genotypes. There is, however, an inherent bias in that those with more severe virilization mostly are the ones with early operation. Even so, many women with lower degrees of virilization were also operated early. Thus, we have no knowledge of how external genitalia change during female puberty and if the natural development possibly creates a better functional and esthetic result than surgery. Moreover, a large proportion did not have a clear opinion making the groups with a personal opinion on early or late surgery even smaller.

The data was collected 20 years ago, before the discussion and questioning of early genital surgery. Hence, the women in this study have not been exposed to the discussion from a human rights perspective and the thoughts about individual self-determination. This makes it difficult to draw conclusions regarding the present situation.

## Conclusion

In this reanalysis of data collected more than 20 years ago, before the questioning of early genital surgery, the timing of surgery was the overarching question. The majority of women with CAH had had genital surgery but almost half (42%) of all participants had no opinion on the timing of surgery. Most of those with an opinion thought that early surgery was preferred. Their preferences were often in accordance with their own age for surgery. This is an extremely difficult subject and for the future it is important to especially follow up the group of non-operated girls with CAH.

## Data Availability

Data is provided within the manuscript.
